# Supplementary material for: The fluid membrane determines mechanics of erythrocyte extracellular vesicles and is softened in hereditary spherocytosis
Source: Nat Commun. 2018 Nov 23;9:4960. doi: 10.1038/s41467-018-07445-x (PMC6251882; doi:10.1038/s41467-018-07445-x)
Supplement: Supplementary file 1 — Supplementary Information [file 41467_2018_7445_MOESM1_ESM.pdf]

SUPPLEMENTARY INFORMATION

The Fluid Membrane determines Mechanics of Erythrocyte Extracellular Vesicles and is Softened in Hereditary Spherocytosis

Daan Vorselen, Susan M. van Dommelen, Raya Sorkin, Melissa C. Piontek, Jürgen Schiller, Sander T. Döpp, Sander A.A. Kooijmans, Brigitte A. van Oirschot, Birgitta A. Versluijs, Marc B. Bierings, Richard van Wijk, Raymond M. Schiffelers, Gijs J.L. Wuite and Wouter H. Roos

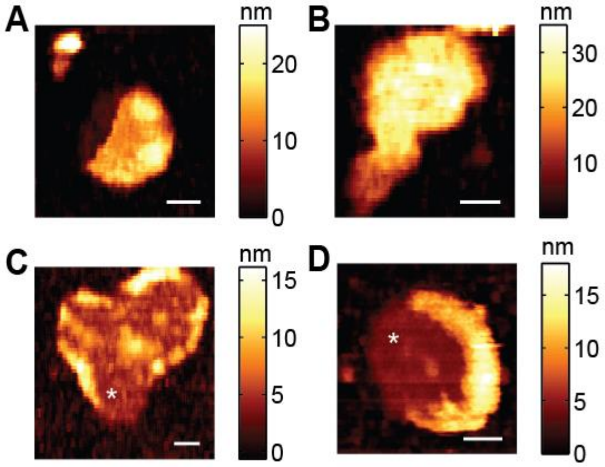

Supplementary Figure 1: Additional structures of collapsed EVs. A-D) AFM topography images showing collapsed EVs. Colour scale indicates height. **A,B)** Flat structures with mean height of about 15 nm respectively 26 nm. **C-D)** Elevated halo-like edges with maximum height 15-20 nm. Panel A, B & C show collapsed EVs from donor 2, panel D shows a collapsed EV from donor 1. The white asterisks indicate areas with the expected height of a supported lipid bilayer (~4 nm). Scale bar length is 50 nm in every panel.

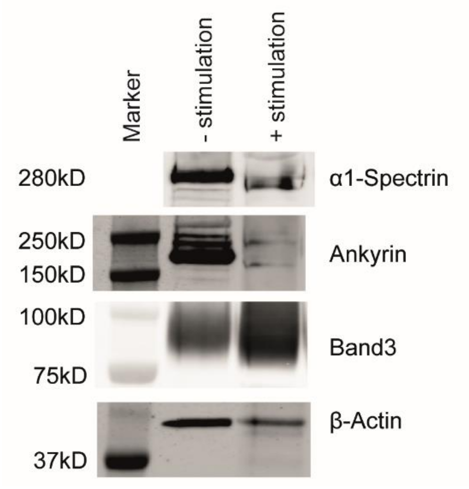

Supplementary Figure 2: Comparison protein composition Ca^{2+} stimulated and non-stimulated RBC EVs. Healthy donor RBCs were either stimulated with calcium ionophore as described before, or were treated identically, but without the addition of calcium ionophore. EV proteins were subjected to electrophoresis. To make a good comparison of key proteins, 3 µg protein of the EVs from unstimulated RBCs was loaded, versus 50 µg protein of the EVs from stimulated RBCs. This was needed, because the last sample contained higher amounts of hemoglobin than the first sample. Apparently, calcium ionophore stimulation leads to EVs with higher amounts of hemoglobin compared with other proteins. In both EV populations, spectrin was present. Marker for α1-spectrin is missing, as it was recorded at a different wavelength (700 nm) than the protein band (800 nm). Full uncropped gels are provided in supplementary figure 8.

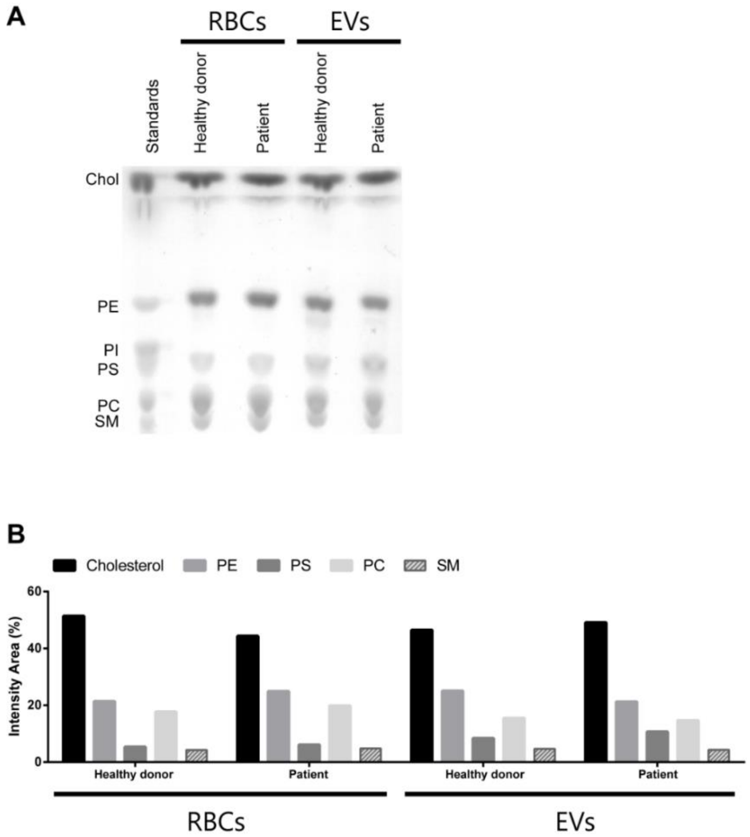

Supplementary Figure 3: Lipid content of RBC EVs. RBC and EV lipids were extracted using the Bligh and Dyer method.[1] **A)** Lipids were subjected to thin-layer chromatography and compared with seven lipid standards. **B)** Chromatogram dots were quantified using custom MATLAB software. Briefly, boxes were drawn around each lane. The sum of intensity along the vertical axis (minus the background observed between the lanes) was then fit using a multimodal (6 modes) Gaussian. The area under the Gaussians was then used to estimate the percentages. The first two Gaussians were taken together, since both correspond to cholesterol. Lipid composition was comparable in all samples. Chol: cholesterol; PE phosphatidylethanolamine; PI: phosphatidylinositol; PS: phosphatidylserine; GM3: monosialodihexosylganglioside 3; PC: phosphatidylcholine; SM: sphingomyelin. Patient data was obtained from the patient with the 4 base pair insertion in *ANK1* that did not undergo splenectomy.

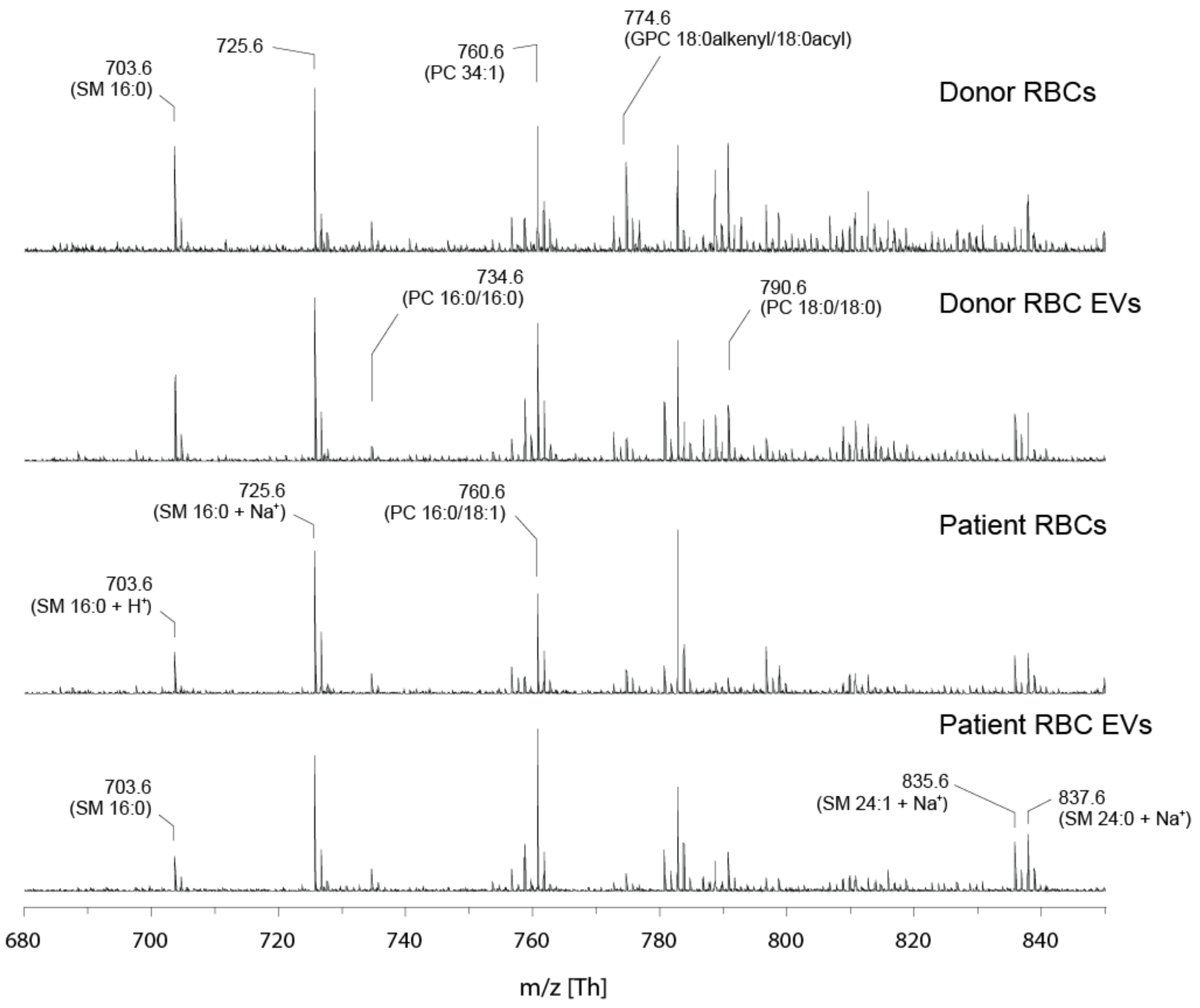

Supplementary Figure 4: Positive ion MALDI-TOF mass spectra of human erythrocyte ghosts (as the control) and EVs. All spectra were obtained with 2,5-dihydroxybenzoic acid (DHB) as the matrix. All peaks are marked by their *m/z* ratios and the most abundant peaks are directly assigned to the corresponding phospholipids. In some cases, both fatty acyl residues in the individual phospholipids were combined because we failed to resolve the individual fatty acyl residues by MS/MS. The differences in peak intensities between the samples are quantified in supplementary table 1. Patient data was obtained from the patient with the 4 base pair insertion in *ANK1* that did not undergo splenectomy. For the raw data see Supplementary Data 1.

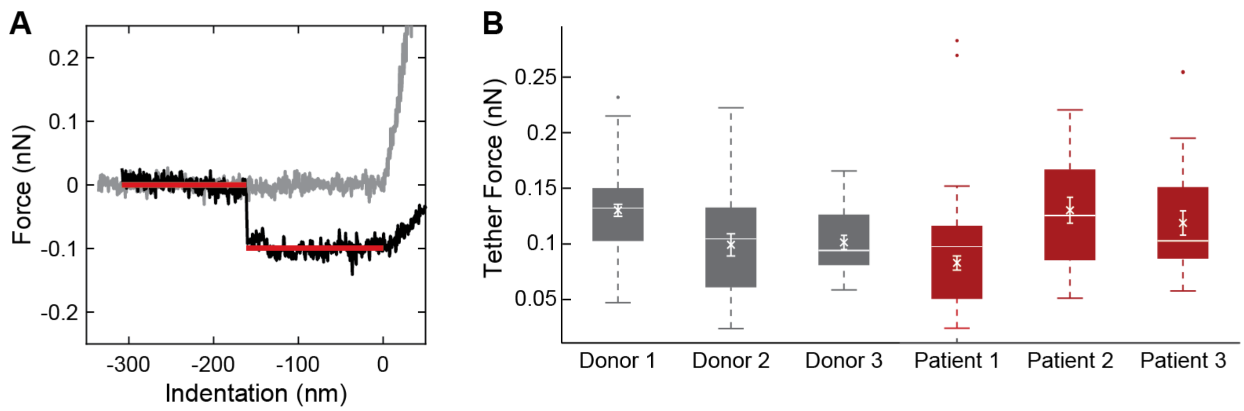

Supplementary Figure 5: Distribution of tether forces observed during retraction of the AFM tip. A) Typical tether formation during an FDC on a single vesicle from donor 1 (approach in grey, retrace in black). Red lines indicate two fitted regimes; the difference is the tether force. **B)** Box plots in which the median is marked by the white line in the box, box limits indicate upper and lower quartiles and whiskers indicate 1.5x interquartile range. White cross and error bars indicate mean and s.e.m. as determined by bootstrapping (1000 repetitions). Tethers were found in ca. 68% of donor 1 EVs (F_t = 130 ± 6 pN, standard error of the mean (s.e.m.), N = 49 tethers), ca. 45% of FDCs from donor 2 EVs (F_t = 100 ± 10 pN, s.e.m., N = 25) and ca. 70% of FDCs from donor 3 EVs (F_t = 100 ± 6 pN, s.e.m., N = 20), ca. 45% of patient 1 EVs (F_t = 80 ± 6 pN (s.e.m., N = 33)), ca. 50% of patient 2 EVs (F_t = 120 ± 5 pN (s.e.m., N = 21)) and ca. 60% of patient 3 EVs (F_t = 130 ± 5 pN (s.e.m., N = 21)). For this and further analysis, outliers with a tether force above 0.25 nN were excluded, since such (rare) events could correspond to pulling a double lipid bilayer tether, as the force is ~2 fold that of the majority of the observed tethers.

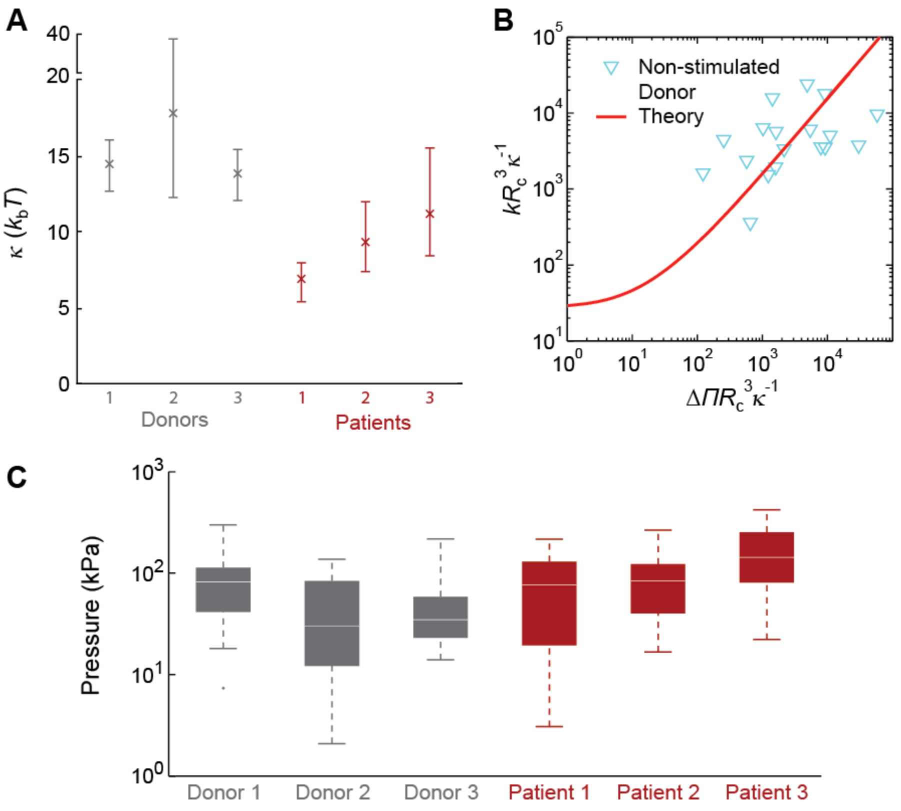

Supplementary Figure 6: Bending modulus and pressure estimates for individual donors and patients. A) Crosses mark the fitted bending modulus estimates from the data sets. The distribution shown in the graphs is provided by bootstrapping, for which samples equal in size to the original sample sizes (N = 49, 25, 20, 33, 21, 21 respectively) were randomly drawn and fitted (1000 times for each condition). Error bars indicate 68% bootstrapping intervals (corresponding to 1 standard deviation for normal distributions). **B)** Dimensionless pressure versus dimensionless stiffness for 18 non-stimulated vesicles from a fourth donor (cyan triangles). Treatment of red blood cells and isolation of vesicles was identical to donor and patient samples, except that no Ca^{2+} ionophore was added to the Ringer's buffer. Theoretical prediction (solid red curve) is based on an adaptation of Canham-Helfrich theory[2,3] and describes mechanical behavior of small fluid vesicles[4]. Data was fitted to the theoretical prediction with the bending modulus κ as single parameter, giving $\kappa = 17$ (13 – 24, 68% c.i. obtained by bootstrapping). **C)** Pressure estimates for donor and patient vesicles. The median is marked by the white line in the box, box limits indicate upper and lower quartiles and whiskers indicate 1.5x interquartile range. Pressure estimates can be made after the bending modulus is determined[4] as $\Delta \Pi = F_{\mathrm{t}}{}^2(4\pi^2 R\kappa)^{-1}$. Number of samples in each conditions is the same as in a.

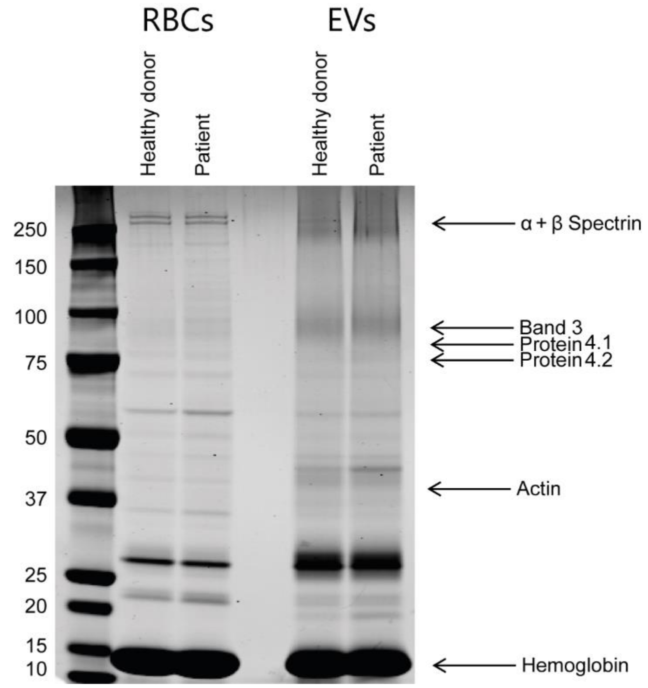

Supplementary Figure 7: Protein content of spherocytosis patient derived RBC EVs. RBC and EV proteins were subjected to SDS-PAGE, by loading 10 µg protein per lane. After running, proteins were stained and protein patterns were compared with patterns of RBC EVs from a healthy donor. Protein patterns found in the patient EVs differ from that found in EVs from a healthy donor, especially around 20 and 40 kDa. Identities of differentially expressed proteins are however unknown. Patient data was obtained from the patient with the 4 base pair insertion in *ANK1* that did not undergo splenectomy.

A Figure 3e uncropped

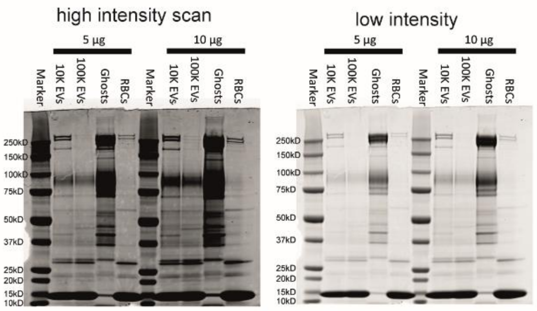

B Figure 5c uncropped

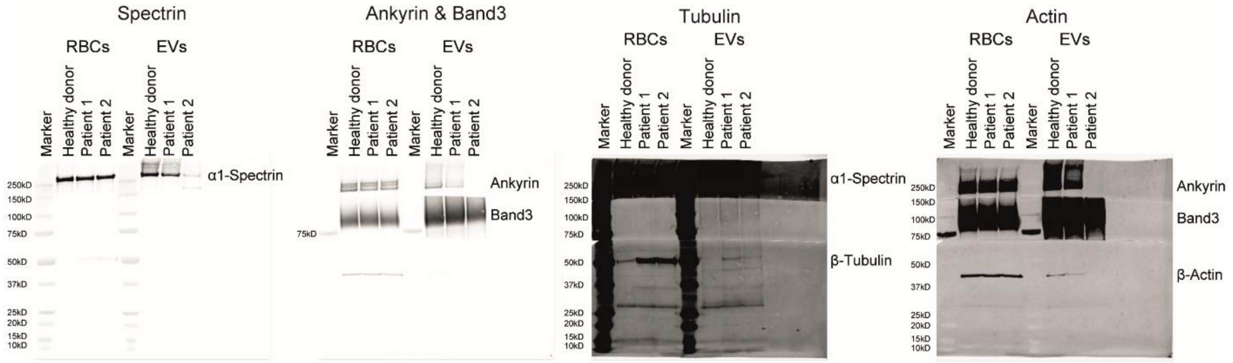

C Supplementary Figure 2 uncropped

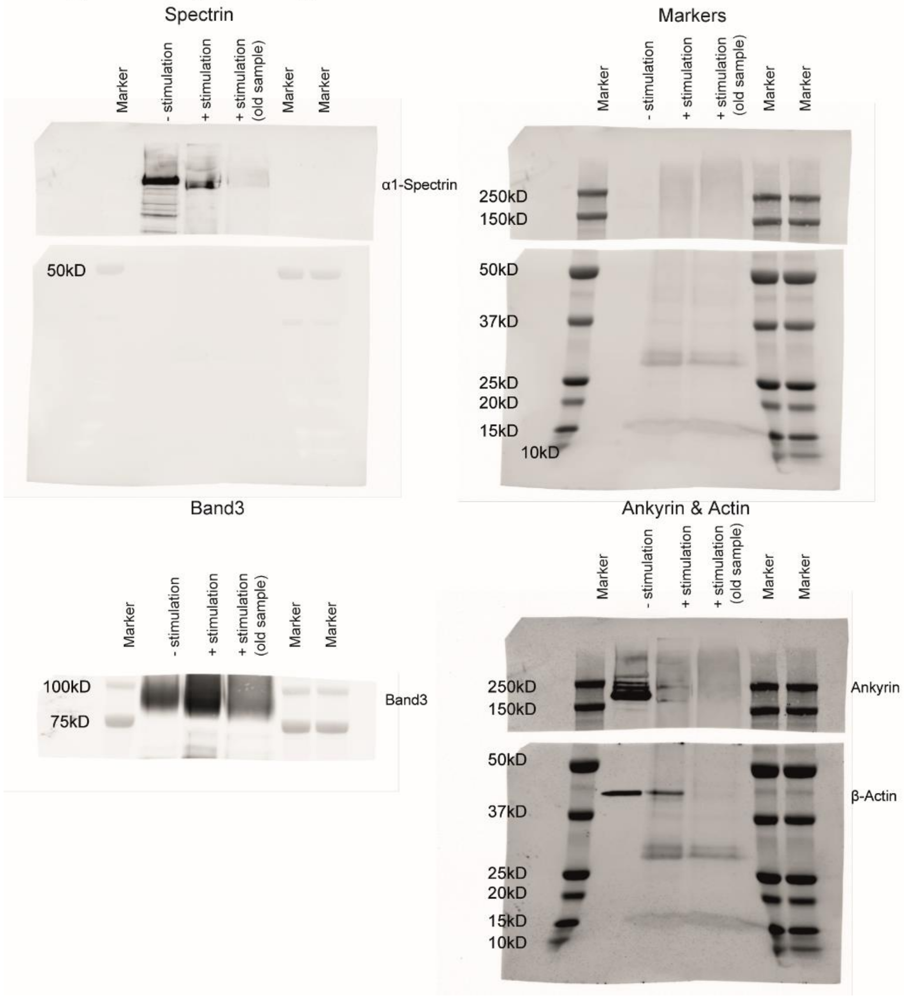

Supplementary Figure 8: Uncropped gels and blots. **A)** EV, ghost and RBC proteins subjected to SDS-PAGE. 100k EVs (EVs obtained after centrifugation at $10^5 \times g$) are presented in figure 3e in the main text. **B)** RBC and EV protein subjected to electrophoresis (see details in figure 5c in the main text). Data from patient 1 (the patient with the 4 base pair insertion in ANK1 that did not undergo splenectomy) is presented in the main text. Patient 2 is the other patient with the 4 base pair insertion in ANK1. The sample from this patient used for gel electrophoresis was taken before splenectomy, whereas all AFM data for this patient were taken after splenectomy. Spectrin & tubulin blots were imaged at 700 nm, whereas the ankyrin & band 3 and the actin blot were imaged at 800 nm. **C)** EV proteins subjected to electrophoresis (see details in supplementary figure 2). All blots were imaged at 700 nm, except for the spectrin blot, which was imaged at 800 nm.

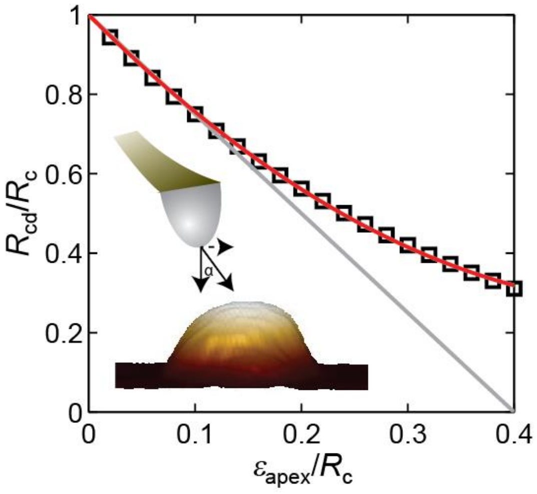

Supplementary Figure 9: Deformation correction. Deformation of the apex versus decrease in the radius of curvature (R_{cd} = deformed radius of curvature), both measured as fraction of the non-deformed radius of curvature R_c (Figure adapted from ref. 4). Data is simulated from a model taking only into account the angle of the applied force (see inset). Since the feedback only measures forces normal to the surface, higher total forces are exerted on the side of the vesicle. The grey line is the linear approximation (slope = 2.5) that we used in previous work[4]. This linear approximation is accurate until apex deformation of ~0.15 R_c. Because some data in this work fell outside this interval (maximum apex deformation ~ 0.3 R_c), here we use a quadratic approximation (red solid line), which was obtained by fitting the simulated data points (black squares): $R_{cd}/R_c = 2.46\,(\varepsilon_{apex}/R_c)^2 - 2.69\,(\varepsilon_{apex}/R_c) + 1$, where ε_{apex} is the deformation at the apex. We performed a correction for the experimentally found radii of curvature based on this quadratic fit. To find the deformation of the center of the vesicle due to imaging forces (ε_{apex}), the height obtained from FDCs was compared with the height obtained from images. For the radius of curvature, we then applied the quadratic correction:

$$R_c = 0.5\left(R_{cd} + 2.69\varepsilon_{apex}{}^2 + \sqrt{\left(R_{cd} + 2.69\varepsilon_{apex}\right)^2 - 9.85\varepsilon_{apex}{}^2}\right),$$

which is obtained by rearranging the relationship found above. We also applied the previously used linear approximation, which did not affect the main conclusions of this work (data not shown).

Supplementary Table 1: **Quantitative comparison of selected lipid species.** Relative moieties (in %) of selected GPC and SM species. All data were obtained from the positive ion MALDI-TOF mass spectra. The intensities of all detected peaks (either GPC or SM) were added and the sum of the proton and sodium adducts of selected lipids divided by this value. Only peaks with at least 0.1% of the intensity of the base peak were used. Only selected species that showed differences between the samples are shown. Patient data was obtained from the patient with the 4 base pair insertion in *ANK1* that did not undergo splenectomy. Also see Supplementary Data 2.

m/z	Donor RBC	Donor RBC EVs	Patient RBC	Patient RBC EVs
734.6/754.6 (PC 2×16:0)	6.3	4.0	8.2	8.5
774.6/796.6 (GPC 18:0alkenyl/18:0acyl)	13.2	5.2	12.4	5.9
790.6/812.6 (PC 2×18:0)	15.9	10.5	6.1	9.3
703.6/725.6 (SM 16:0)	66.5	65.8	70.8	53.2
813.6/835.6 (SM 24:1)	12.4	17.7	15.4	19.7
815.6/837.6 (SM 24:0)	21.2	16.4	15.6	24.8

Supplementary Table 2: Contribution of reticulocytes to the isolated EV population. In order to separate RBCs based on age, healthy donor RBCs were subjected to a Percoll gradient. Three cell populations were isolated: reticulocytes (on top of 40% Percoll layer), young RBCs (on top of 59% Percoll layer) and mature RBCs (below 65% Percoll layer). Using vital staining the reticulocyte number in each fraction was determined and showed that the reticulocyte fraction contained 75-80% reticulocytes, while in the total RBC population this was 1-2%. The three fractions and a sample of the total RBC population were stimulated with calcium ionophore and EVs were isolated. Using NTA, particle numbers were determined and calculated to EVs released per cell. The excretion of EVs by reticulocytes was ~300 fold lower, and hence even for patients with high reticulocyte counts (up to 20%), less than 0.1% of EVs will be reticulocyte derived.

	Total RBCs	Reticulocytes	Young RBCs	Mature RBCs
EVs released per cell	256	1	11	342

Supplementary References

1. Bligh, E. G. & Dyer, W. J. A Rapid Method of Total Lipid Extraction and Purification. *Can. J. Biochem. Physiol.* **37,** 911–917 (1959).

2. Helfrich, W. Elastic properties of lipid bilayers: theory and possible experiments. *Z. Naturforsch. C.* **28,** 693–703 (1973).

3. Canham, P. B. The minimum energy of bending as a possible explanation of the biconcave shape of the human red blood cell. *J. Theor. Biol.* **26,** 61–81 (1970).

4. Vorselen, D., MacKintosh, F. C., Roos, W. H. & Wuite, G. J. L. Competition between Bending and Internal Pressure Governs the Mechanics of Fluid Nanovesicles. *ACS Nano* **11,** 2628–2636 (2017).
