# Supplementary material for: The fluid membrane determines mechanics of erythrocyte extracellular vesicles and is softened in hereditary spherocytosis
Source: Nat Commun. 2018 Nov 23;9:4960. doi: 10.1038/s41467-018-07445-x (PMC6251882; doi:10.1038/s41467-018-07445-x)
Supplement: Supplementary file 3 — Description of Additional Supplementary Files [file 41467_2018_7445_MOESM3_ESM.pdf]

Description of Additional Supplementary Files

File Name: Supplementary Data 1

Description: Positive ion MALDI-TOF mass spectra. Spectra for donor RBCs, donor RBC EVs, patient RBCs, and patient RBC EVs, where patient data was obtained from the patient with the 4 base pair insertion in ANK1 that did not undergo splenectomy. All spectra were obtained with 2,5-dihydroxybenzoic acid (DHB) as the matrix.

File Name: Supplementary Data 2

Description: Positive ion MALDI-TOF peak annotations. Assignment of the m/z values detected in the positive ion MALDI-TOF mass spectra of human erythrocyte ghosts (as the control) and EVs. All spectra were recorded with 2,5-dihydroxybenzoic acid (DHB) as the matrix which leads to the generation of both, the proton and the sodium adducts. Please note that only major signals are assigned, and minor peaks are neglected: due to the small signal intensities MS/MS spectra could not be recorded and, therefore, assignments would be ambiguous. The presence of alkenyl-acyl compounds ("plasmalogens") was confirmed by the treatment with diluted HCl since plasmalogens are extremely sensitive to acids and decay under the generation of the corresponding lysophosphatidylcholines (LPCs)
