# Supplementary material for: The fluid membrane determines mechanics of erythrocyte extracellular vesicles and is softened in hereditary spherocytosis
Source: Nat Commun. 2018 Nov 23;9:4960. doi: 10.1038/s41467-018-07445-x (PMC6251882; doi:10.1038/s41467-018-07445-x)
Supplement: Supplementary file 6 — Reporting Summary [file 41467_2018_7445_MOESM6_ESM.pdf]

nature research

Corresponding author(s): Wouter H Roos, Gijs JL Wuite

Reporting Summary

Nature Research wishes to improve the reproducibility of the work that we publish. This form provides structure for consistency and transparency in reporting. For further information on Nature Research policies, see Authors & Referees and the Editorial Policy Checklist.

Statistical parameters

When statistical analyses are reported, confirm that the following items are present in the relevant location (e.g. figure legend, table legend, main text, or Methods section).

n/a	Confirmed	
☐	☒	The exact sample size (*n*) for each experimental group/condition, given as a discrete number and unit of measurement
☐	☒	An indication of whether measurements were taken from distinct samples or whether the same sample was measured repeatedly
☐	☒	The statistical test(s) used AND whether they are one- or two-sided<br>*Only common tests should be described solely by name; describe more complex techniques in the Methods section.*
☒	☐	A description of all covariates tested
☒	☐	A description of any assumptions or corrections, such as tests of normality and adjustment for multiple comparisons
☐	☒	A full description of the statistics including central tendency (e.g. means) or other basic estimates (e.g. regression coefficient) AND variation (e.g. standard deviation) or associated estimates of uncertainty (e.g. confidence intervals)
☐	☒	For null hypothesis testing, the test statistic (e.g. *F*, *t*, *r*) with confidence intervals, effect sizes, degrees of freedom and *P* value noted<br>*Give P values as exact values whenever suitable.*
☒	☐	For Bayesian analysis, information on the choice of priors and Markov chain Monte Carlo settings
☒	☐	For hierarchical and complex designs, identification of the appropriate level for tests and full reporting of outcomes
☒	☐	Estimates of effect sizes (e.g. Cohen's *d*, Pearson's *r*), indicating how they were calculated
☐	☒	Clearly defined error bars<br>*State explicitly what error bars represent (e.g. SD, SE, CI)*

Our web collection on statistics for biologists may be useful.

Software and code

Policy information about availability of computer code

Data collection	For collection of the AFM data NanoScope Software (8) by Bruker was used.
Data analysis	The authors declare that the custom (Matlab) code used for analyzing the data in this study is available from the authors upon reasonable request.

For manuscripts utilizing custom algorithms or software that are central to the research but not yet described in published literature, software must be made available to editors/reviewers upon request. We strongly encourage code deposition in a community repository (e.g. GitHub). See the Nature Research guidelines for submitting code & software for further information.

Data

Policy information about availability of data

All manuscripts must include a data availability statement. This statement should provide the following information, where applicable:

- Accession codes, unique identifiers, or web links for publicly available datasets
- A list of figures that have associated raw data
- A description of any restrictions on data availability

The authors declare that the data supporting the findings of this study are available within the article (and its Supplementary Information) or available from the authors upon request.

Field-specific reporting

Please select the best fit for your research. If you are not sure, read the appropriate sections before making your selection.

☒ Life sciences ☐ Behavioural & social sciences ☐ Ecological, evolutionary & environmental sciences

For a reference copy of the document with all sections, see nature.com/authors/policies/ReportingSummary-flat.pdf

Life sciences study design

All studies must disclose on these points even when the disclosure is negative.

Sample size	No sample size calculation was performed beforehand. Sample sizes (3 donors and 3 patients) for the main comparison in the paper were based on the minimum required for proper statistical testing, because of the difficulty in obtaining data of this sort.
Data exclusions	All relevant data exclusions are described in the manuscript. In summary:<br>- vesicles that appear higher on the image than during force distance curves were excluded since this may indicate off-centre indentation<br>- vesicles for which we could not measure a tether force were excluded, as we can not estimate their internal pressure<br>- vesicles with tether force > 250 pN were excluded as this may correspond to a double tether (as the force is approximately 2-fold that of the most common tether forces)
Replication	We included all patient and donor samples measured in the manuscript, and there are no findings that could not be replicated/reproduced. As decribed in the manuscript, experiments were performed with 3 donors and 3 patients, and a two sided two sample t-test indicated that there is a significant difference between donor and patient samples. Additionally, at least 20 vesicles were measured for each donor/patient sample.
Randomization	Randomization was not relevant, since the two experimental groups were based on predetermined characteristics: hereditary spherocytosis patients and healthy donors.
Blinding	Investigators were not blinded to group allocation. Firstly, this would have been unfeasible as patient samples could only be obtained occasionally and experiments needed to be planned accordingly since sample measurements needed to be done directly and took a full week. Secondly, experiments are done on a vesicle-by-vesicle basis and analysis was multifactorial (tether force, stiffness, size) and mostly automated. Due to the the multifactorial analysis, it is far from obvious how selecting/excluding specific vesicles will affect bending modulus measurements, making blinding less relevant.

Reporting for specific materials, systems and methods

Materials & experimental systems

n/a	Involved in the study
☒	☐ Unique biological materials
☐	☒ Antibodies
☒	☐ Eukaryotic cell lines
☒	☐ Palaeontology
☒	☐ Animals and other organisms
☐	☒ Human research participants

Methods

n/a	Involved in the study
☒	☐ ChIP-seq
☒	☐ Flow cytometry
☒	☐ MRI-based neuroimaging

Antibodies

Antibodies used	The following commercial primary antibodies for western blot were used:<br>- Anti-alpha 1 Spectrin (ab139403, Abcam),<br>- Anti-beta Tubulin (ab6046, Abcam)<br>- Anti-Band 3 (B9277, Sigma),<br>- Anti-beta-actin (3700, Cell Signaling Technology)<br>- Anti-Ankyrin-1 (9473PA, IBGRL)<br>The following commercial secondary antibodies were used:<br>- Goat anti-Rabbit (926-32211, LI-COR)<br>- Donkey anti-Mouse (926-32212, LI-COR)<br>- Goat anti-Mouse (A-21057, ThermoFisher Scientific)<br>- Goat anti-Rabbit (A-21076, ThermoFisher Scientific)
Validation	The antibodies used for immunofluorescence were validated by the suppliers. Specifically:

- Specificity of Anti-alpha 1 Spectrin (ab139403, Abcam) and Anti-beta Tubulin (ab6046, Abcam) were validated using cell lysates (k562, and Hela, A431, MCF7 & 293 respectively. Abcam also cites >400 papers for the anti-beta tubulin antibody.
- Anti-Band 3 (B9277, Sigma). Specificity was checked using Human erythrocytes ghosts extracts. Sigma also cites the following papers for use in western blots: Dupuy & Engelman, PNAS 105: 2848 (2008); Govekar et al, Anemia: 168050 (2012)
- Anti-beta-actin (3700, Cell Signaling Technology) specificity was validated using cell extracts of 5 different cell lines. Cell signaling technology also cites over 650 papers that have used the antibody.
- Anti-Ankyrin-1 (9473PA, IBGRL) specificity was validated using partial purified erythrocyte membrane preparations. IBGRL also cites: Bell et al, Plos One 8: e60300 (2013)

Human research participants

Policy information about studies involving human research participants

Population characteristics	Population characteristics of healthy donors are unknown.
Recruitment	Patient blood was collected during regular controls of Hereditary Spherocytosis patients due to Ankyrin deficiency. After the study was conceived, data was collected on the first incoming patients that gave informed consent.
